# Supplementary material for: Coordination of cell cycle and morphogenesis during organ formation
Source: eLife. 2024 Jan 26;13:e95830. doi: 10.7554/eLife.95830 (PMC10869137; doi:10.7554/eLife.95830)
Supplement: Supplementary file 1. [file elife-95830-supp1.docx]

**Supplementary file 1. Fly strains used**

| **Fly strain** | **Sources and References** | **RRID** |
| --- | --- | --- |
| *Oregon R* (wild type) |  |  |
| *fkh-Gal4* | Henderson and Andrew, 2000 | BDSC_78060 |
| *hkb^121^* | This study |  |
| *hkb^131^* | This study |  |
| *hkb^351^* | This study |  |
| *hkb^391^* | This study |  |
| *Hkb^WT^-mCh* | This study |  |
| *Hkb^mut^-mCh* | This study |  |
| *hkb^2^* | Weigel et al., 1990 | BDSC_5457 |
| *hkb^A321R1^* | Gaul and Weigel, 1991 |  |
| *UAS-p35* |  | BDSC_5072 |
| *UAS-Hkb* | Myat and Andrew, 2002 |  |
| *CycE^AR95^* |  | BDSC_6637 |
| *CycD^1^* |  | BDSC_55125 |
| *CycA^C8LR1^* |  | BDSC_6627 |
| *Cdk1^B47^* |  | BDSC_6643 |
| *TRiP.GL00511 (UAS-CycE RNAi)* |  | BDSC_36092 |
| *TRiP.HMS01531* (*UAS*-*Cdk1 RNAi*) |  | BDSC_36117 |
| *UAS-Fzr* |  | BDSC_91688 |
| *Ubi-GFP.E2f1, Ubi-mRFP1.CycB* |  | BDSC_55098 |
