# Supplementary material for: Coordination of cell cycle and morphogenesis during organ formation
Source: eLife. 2024 Jan 26;13:e95830. doi: 10.7554/eLife.95830 (PMC10869137; doi:10.7554/eLife.95830)
Supplement: Supplementary file 2. [file elife-95830-supp2.docx]

**Supplementary file 2. Antibodies Used**

| **Antibody** | **Source** | **RRID** | **Dilution** |
| --- | --- | --- | --- |
| α-Ecad (rat) | DSHB (DCAD2) | AB_528120 | 1:50 |
| α-CrebA (rat) | Andrew lab (John Hopkins University); re-made in this study |  | 1:3,000 |
| α-CrebA (rabbit) | Andrew lab (John Hopkins University) | AB_10805295 | 1:5,000 |
| α-CrebA (guinea pig) | This study |  | 1:5,000 |
| α-GFP (mouse) | Invitrogen (A11120) | AB_221568 | 1:500 |
| α-GFP (chicken) | Invitrogen (A10262) | AB_2534023 | 1:1,000 |
| α-RFP (rabbit) | Invitrogen (R10367) | AB_10563941 | 1: 2,000 |
| α-β-galactosidase (rabbit) | Invitrogen (A11132) | AB_221539 | 1:500 |
| α-β-galactosidase (mouse) | Invitrogen (MA5-15222) | AB_10980770 | 1:500 |
| α-mCherry (rat) | Invitrogen (M11217) | AB_2536611 | 1:1,000 |
| α-mCherry (rabbit) | Invitrogen (PA534974) | AB_2552323 | 1:1,000 |
| α-DCP-1 (rabbit) | Cell signaling technology (9578) | AB_2721060 | 1:200 |
| α-phosphohistone 3 (rabbit) | Invitrogen (PA517869) | AB_10984484 | 1:1,000 |
| α-tyrosinated α-tubulin (rat) | Invitrogen (MA1-80017) | AB_2210201 | 1:1,000 |
| α-H2AvD (mouse) | DSHB (UNC93-5.2.1) | AB_2618077 | 1: 1,000 |
| α-Sage (guinea pig) | Andrew lab (Johns Hopkins University) |  | 1: 100 |
| α-CyclinB (mouse) | DSHB (F2F4) | AB_528189 | 1: 5 |
| α-Crb (mouse) | DSHB (Cq4) | AB_528181 | 1:10 |
| α-Rab11 (rabbit) | Andrew lab (John Hopkins University) |  | 1:500 |
| α-mCherry (rat) | Invitrogen (M11217) | AB_2536611 | 1:1,000 |
| α-mCherry (rabbit) | Invitrogen (PA534974) | AB_2552323 | 1:1,000 |
| α-α-Spectrin (mouse) | DSHB (3A9) | AB_528473 | 1:2 |
| α-Dead Ringer (chicken) | Andrew lab (Johns Hopkins University) |  | 1:1000 |
| Phalloidin Alexa 488 | Invitrogen (A12379) | AB_2759222 | 1:250 |
| Alexa Fluor 488/568/647- coupled secondary antibodies | Invitrogen |  | 1:500 |
